# Influenza Outbreak during Sydney World Youth Day 2008: The Utility of Laboratory Testing and Case Definitions on Mass Gathering Outbreak Containment

**DOI:** 10.1371/journal.pone.0006620

**Published:** 2009-09-03

**Authors:** Sebastiaan J. van Hal, Hong Foo, Christopher C. Blyth, Kenneth McPhie, Paul Armstrong, Vitali Sintchenko, Dominic E. Dwyer

**Affiliations:** 1 Centre for Infectious Diseases and Microbiology, Institute of Clinical Pathology & Medical Research, Westmead Hospital, Westmead, Sydney, New South Wales, Australia; 2 Western Clinical School, University of Sydney, Westmead, Sydney, New South Wales, Australia; 3 Biopreparedness Unit, NSW Department of Health, North Sydney, New South Wales, Australia; BMSI-A*STAR, Singapore

## Abstract

**Background:**

Influenza causes annual epidemics and often results in extensive outbreaks in closed communities. To minimize transmission, a range of interventions have been suggested. For these to be effective, an accurate and timely diagnosis of influenza is required. This is confirmed by a positive laboratory test result in an individual whose symptoms are consistent with a predefined clinical case definition. However, the utility of these clinical case definitions and laboratory testing in mass gathering outbreaks remains unknown.

**Methods and Results:**

An influenza outbreak was identified during World Youth Day 2008 in Sydney. From the data collected on pilgrims presenting to a single clinic, a Markov model was developed and validated against the actual epidemic curve. Simulations were performed to examine the utility of different clinical case definitions and laboratory testing strategies for containment of influenza outbreaks. Clinical case definitions were found to have the greatest impact on averting further cases with no added benefit when combined with any laboratory test. Although nucleic acid testing (NAT) demonstrated higher utility than indirect immunofluorescence antigen or on-site point-of-care testing, this effect was lost when laboratory NAT turnaround times was included. The main benefit of laboratory confirmation was limited to identification of true influenza cases amenable to interventions such as antiviral therapy.

**Conclusions:**

Continuous re-evaluation of case definitions and laboratory testing strategies are essential for effective management of influenza outbreaks during mass gatherings.

## Introduction

Seasonal influenza is a contagious, acute febrile respiratory illness which causes annual global epidemics and is associated with significant morbidity and mortality. This seasonal activity often results in extensive outbreaks following introduction into closed communities such as aged care facilities, schools, cruise ships or military barracks [1], [2], [3]. These outbreaks can be associated with significant consequences especially in aged care facilities. A range of interventions has been suggested to minimize transmission such as social distancing, improved hygiene, mass vaccination and antiviral treatment/prophylaxis [4].

The effectiveness of these interventions is reliant on the accurate and timely diagnosis of influenza. The first component is identifying individuals who require laboratory testing to confirm the clinical diagnosis. This occurs when an individual's symptoms are consistent with a predefined clinical case definition. The utility of the applied case definition (i.e. sensitivity) is influenced by various factors including the age distribution of affected populations and the location of an outbreak [5], [6], [7], [8].

Following identification of an influenza-like illness (ILI) case, laboratory testing is generally employed to confirm the clinical diagnosis. The specific laboratory test chosen depends on several factors including accuracy, turn-around-time (TAT), availability, cost and phase of the outbreak. During the early and/or pre-outbreak stages, laboratory testing aimed at surveillance and influenza case identification are required thus highly sensitive nucleic acid tests (NAT) and virus isolation are usually preferred. In contrast, when laboratory testing is used during the containment phase, the TAT of testing becomes the most important consideration to maximize benefits with point-of-care antigen tests (POCT) or NAT testing fulfilling this role.

Unlike data from closed community, data from clusters of ILI outbreaks during mass gatherings, such as religious celebrations and large community events, are seldom collected [9]. Therefore, no consensus exists on the optimal ILI case definition to assist in the containment of mass gatherings outbreaks [10]. Similarly, the relative utility of different laboratory testing strategies during a mass gathering outbreaks have not been studied.

World Youth Day (WYD) is a large Catholic youth festival instituted by Pope John Paul II in 1986 which is held in different locations every 2–3 years. In 2008, WYD was held in Sydney, Australia, from the 15 to 20 of July (http://www.wyd2008.org). WYD festivities commenced with cultural exchange events between different Dioceses across Australia. Following this pilgrims converged on Sydney for WYD celebrations which officially commenced on the 15^th^ July [11]. An estimated 223,000 pilgrims from 170 different countries attended a series of mass gatherings culminating in the Papal Mass on July 20.

During WYD, outbreaks of ILI due to influenza A and influenza B viruses amongst pilgrims were identified. Using prospectively collected data, modeling allowed us to explore the impact of various clinical case definitions and laboratory testing strategies in the containment of influenza during mass gatherings, and to offer new insights into epidemic testing strategies.

## Materials and Methods

### Accommodation during WYD

Approximately 100,000 pilgrims requested accommodation from WYD organizers and were assigned to specific venues across metropolitan Sydney [11]. These served as “home” bases with pilgrims traveling during the day to attend the various large outdoor religious gatherings with other pilgrims. Venues included school halls, dormitories, and covered sporting arenas. The largest venue was the Sydney Olympic Park Site (SOPS) (location of the Olympic Games in 2000); housing anywhere between 6000 and 12000 pilgrims each night. Pilgrims were allocated floor space and slept on temporary floor mats with access to shared toilets and other facilities. Pilgrims were able to take up residency 3 days prior to the official WYD opening and had to vacate 3 days following the final Papal Mass.

### WYD Influenza Outbreak

An influenza outbreak was first detected and confirmed at one of the alternate accommodation venue. The SOPS clinic was promptly established on the 16^th^ July following identification of pilgrims with symptoms consistent with an ILI. The SOPS clinic was closed on the 23^rd^ July following closure of the SOPS as an accommodation venue.

**Figure 1:**
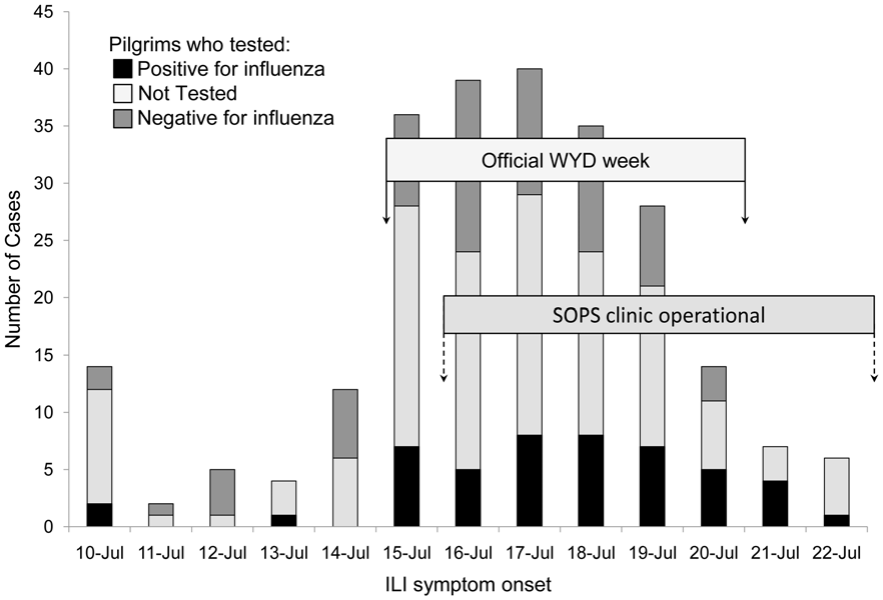
The epidemic curve, as of symptom onset date, from the SOPS clinic. Presenting pilgrims are represented as influenza test positive or negative and not tested.

The subsequently obtained epidemic curve (Figure 1) identifies the date of onset of symptoms from all pilgrims presenting the SOPS clinic.

### Intervention

Pilgrims confirmed as positive for influenza by on-site POCT were prescribed oseltamivir and were voluntarily quarantined with other symptomatic pilgrims separate from the other pilgrims. For the first 48 hours, symptomatic pilgrims were requested to stay on site and not go to the mass events.

### Data collection

Data was prospectively collected on all pilgrims presenting to the SOPS influenza clinic and included demographics, symptoms and body temperature at presentation. Nasal and throat swabs were obtained for laboratory testing. Initial testing was performed by the on-site clinic staff using a point-of-care test (Quickvue A & B®, Quidel Corp., San Diego, CA, USA). Additional nasal swabs were taken and sent to a virology reference laboratory for antigen detection using an indirect fluorescent antibody (IFA) test (Chemicon, International, Inc, Temecula, CA, USA). All positive IFA samples were inoculated into Madin-Darby Canine Kidney (MDCK) cell lines with cultured viral identification confirmed by IFA. Following the outbreak, virus detection was conducted on all samples by polymerase chain reaction (PCR) targeting the matrix region of the influenza virus genome [12], [13]. Testing was performed on the stored respiratory specimen aliquots (at −80°C) following nucleic acid extraction with a commercial assay (Roche® High Pure, Mannheim, Germany).

### Performance of diagnostic techniques and influenza-like illness case definitions

The laboratory “gold standard” was defined as either isolation of influenza virus by culture and/or detection of virus by PCR. The performance of IFA and POCT were assessed against the “gold standard” with PCR sensitivity calculated against viral culture alone. Similarly, the performance sensitivities of the SOPS Outbreak case definition (a history of fever and cough), and various published case definitions were calculated. The published case definitions examined included the Centers for Disease Control (CDC) seasonal surveillance influenza case definition (temperature ≥37.8°C, a cough or sore throat) [14] and the New South Wales Department of Health (NSW) influenza case definition (temperature >38°C, a cough and one other ILI symptom) [15]. In addition, an optimal ILI case definition with maximal sensitivity was formulated based on our data.

### Model

**Figure 2:**
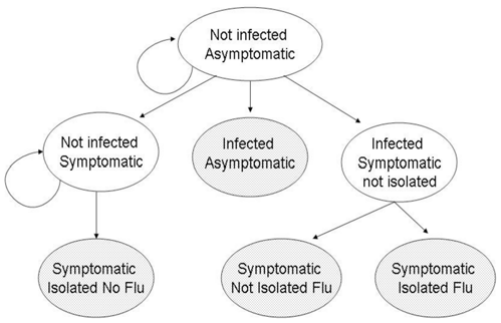
Seven state Markov diagram. Each day of an outbreak a hypothetical cohort of pilgrims moves (following the arrows) to another or returns to the same health state based on transition probabilities. Shaded boxes represent terminal (or absorbing) states.

The impact of the various clinical case definitions, with or without a laboratory test, was examined in a Markov chain model constructed using TreeAge Professsional Software (version 1.5.2, TreeAge Pro, Williamstown, MA, USA) [16]. An individual's infection course (Figure 2) was represented as a sequence of transitions between seven mutually exclusive health states, based on transition probabilities according to published evidence and observations calculated from the recorded WYD influenza outbreak (Table 1). Of the seven possible states, 3 were considered transient (*Not-Infected-Asymptomatic, Not-Infected-Symptomatic* and *Infected-Symptomatic-Not-Isolated*), with the remaining four being terminal (*Infected-Asymptomatic*, *Symptomatic-Isolated-Flu, Symptomatic-Not-Isolated-Flu* and *Symptomatic-Isolated-No-Flu*). Thus, based on the probability of fulfilling the case definition and/or returning a positive laboratory test result, a symptomatic individual with influenza (*Infected-Symptomatic-Not-Isolated*) was then either isolated (*Symptomatic-Isolated-Flu*) or remains infected but not isolated (*Symptomatic-Not-Isolated-Flu*). If not isolated, these individuals were able to infect further susceptible (*Not-Infected-Asymptomatic*) individuals.

**Table 1:** Demographics, ILI symptoms and performance of case definitions and laboratory tests for 242 pilgrims presenting to the Olympic Park Site influenza clinic.

Number of pilgrims screened	242
Sex, male	90 (37%)
Age in years, mean (range)	26 (12–66)
Mean duration of symptoms in days (range)	3 (0–15)
Prior influenza vaccination	24 (10%)
Pilgrims undergoing respiratory sampling	114 (47%)
Pilgrims positive for influenza	45 (19%)
**Symptoms in pilgrims positive for influenza:**	
Temperature >38°C	14 (31%)
History of fever	30 (66%)
Cough	31 (69%)
Sore Throat	31 (69%)
Coryza	32 (71%)
**Number of influenza positive pilgrims meeting a case definition** *	
NSW: Presence of a temperature >38°C, a cough and one other ILIsymptom	12 (26%)
CDC: Presence of temperature >37.8°C, a cough or sore throat	18 (40%)
Outbreak Case definition (history of fever and cough)	30 (66%)
Presence of coryza with or without any other symptom	32 (71%)
**Performance of laboratory Testing** †	
PCR	42 (93%)^1^
Indirect Fluorescent Antibody	33 (74%)^1^
On-site Point-of-Care	26 (56%)^1^

* Case definition outbreak values were subsequently used in the Markov model and are similar to previous published case definition ranges of between 44 and 87% [28], [29], [30], [31], [32].

† The “gold standard” was defined as isolation of influenza virus by culture and/or detection of virus by PCR

^1^The calculated laboratory testing values were subsequently used in the Markov model and are similar to published PCR, IFA and POCT sensitivities of 90 to 100% [13], [33], [34], [35]; 60 to 100% [12], [36], [37] and 50 to 95% [38], [39], [40] respectively.

### Model assumptions

Influenza characteristics adopted in the model were based on previously published observations [17], [18], [19], [20], [21], [22] (Table 2). These include: (1) an incubation period of 48 hours following exposure; (2) the infectivity of an influenza case remained constant over an entire infectious period of 96 hours after which the individual was considered fully immune; (3) Two thirds of individuals with influenza develop symptoms (i.e. a symptomatic illness); (4) The remaining one third of individuals continued to be asymptomatic; and (5) asymptomatic influenza cases were half as infectious as a symptomatic influenza case.

**Table 2:** Baseline probability values and ranges (%) of variables used to construct the Markov model.

Adopted Influenza characteristics	Model value	Published ranges and references
Influenza incubation period	48 hrs	24–96 hrs [17], [18], [19], [20]
Infectious period	96 hrs	24–144 hrs [17], [18], [19], [20]
Probability of a symptomatic infection	0.67	0.67 [17], [24], [41]
Probability of an asymptomatic infection	0.33	0.33 [17], [24], [41]
An asymptomatic infection is half as infectious as a symptomatic infection	^1^/_2_	^1^/_2_ [17], [24], [41]
Number of secondary influenza cases from an index case (Ro)	4.0	1.2–20 [21], [22]
Exposure probability #	0.001–1.0	<0.001–1.0 [42]

* The sensitivity of the case definition or laboratory test is based on the observed influenza outbreak compared to the “gold standard” (see text for details) and corresponds with value used in the Model simulations.

# The number of exposed pilgrims on a particular day divided by the total susceptible population. In turn, the susceptible population is the total population excluding all pilgrims previously infected (and so regarded as immune) and those currently infected with influenza.

Other assumptions were adopted to maximize the utility of clinical case definitions and/or laboratory testing with 1) All symptomatic cases presenting to the clinic on the first day of symptoms. 2) All cases were isolated (100%) when an individual's symptoms met the case definition and/or had a positive test result. An exception was for simulations where TAT for PCR results were included, infected individuals continued exposing asymptomatic individuals pending the PCR result (i.e. after 6, 12, 18 or 24 hours) followed by a 100% isolation rate.

A minimum and maximum attack rates were estimated using data obtained from the outbreak and from these data the model starting prevalence was established. Subsequent probability of being exposed to an influenza case (exposure probability) was calculated and equals the number of exposed pilgrims divided by the total susceptible population (i.e. the total population excluding all pilgrims previously infected and thus regarded as immune, and those currently infected with influenza). The experimental epicurve was compared with the observed epicurve and from this, the reproduction rate (R_0_: the expected number of secondary cases occurring from one infected individual) was estimated [23].

### Simulations

Simulations were performed on a set of 6,000 influenza naïve (non-immune) individuals for 17 equal one-day increments. Seventeen days was selected as this i) corresponded to exhaustion of the outbreak when no intervention is undertaken ii) was inclusive of the total duration of outbreak observed and iii) was similar to the expected duration of other mass events [8]. An initial population size of 6,000 was used as this reflected the core population at the SOPS. Further simulations were performed on a population of 12,000 (the upper limit of the number of pilgrim accommodated at the SOPS), 50,000, 100,000, 223,000 (the total number of pilgrims attending WYD), 500,000 and one million individuals. The number of symptomatic cases at the start of each experiment remained the same irrespective of the population size.

### Ethics Statement

The outbreak was an acute public health problem requiring a rapid response, thus a formal submission for ethics approval was not undertaken. Both data collection and respiratory tract sampling was performed as standard of care. All presenting pilgrims gave verbal consent for both the data collection and respiratory tract sampling. Written consent was not deemed necessary as presentation to the clinic was totally voluntary and involved standard medical practice. The clinical data was subsequently de-identified for analysis.

## Results

### Performance of case definitions and laboratory tests

Of the 242 patients who presented to the SOPS clinic with ILI symptoms, 114 (47%) underwent diagnostic sampling of which 45 (45/114; 41%) were positive for influenza. Symptoms were present for a mean of 3 days (range 0–15 days) with coryza the most common symptom present (32/45; 71%). Fever at time of presentation was present in the minority of patients (14/45; 31%) (Table 1).

The performance of the various published case definitions against the laboratory “gold standard”, was suboptimal with the CDC influenza surveillance and NSW case definitions demonstrating sensitivities of 40% and 26% respectively. The SOPS Outbreak case used during the WYD outbreak had a sensitivity of 66%. The presence of coryza (with or without any other symptom) demonstrated a sensitivity of 71% in our population. The sensitivity of IFA and on-site POCT was 74% and 56% respectively with PCR only 93% (42/45) sensitive as 3 samples were PCR negative but influenza culture positive (Table 1).

### Development and validation of the model

As all presenting pilgrims were not tested, a minimum and maximum attack rate was estimated. The minimum attack rate assumed that all non-tested pilgrims, were not infected (45/242; 19%). The maximum attack rate assumed that all non-tested pilgrims were infected (173/242; 71%). The likely attack rate assumed that the proportion of non-tested pilgrims was the same as those tested (95/242; 39%). Fourteen patients' symptoms started on the 10^th^ of July, of which, only 2 of 4 tested had influenza. Using the above calculated attack rates, the number of expected cases with symptoms on this day ranged between 2 and 9 cases. The model estimated prevalence was assumed to be 0.1% (6/6000) and was applied to all simulations.

**Figure 3:**
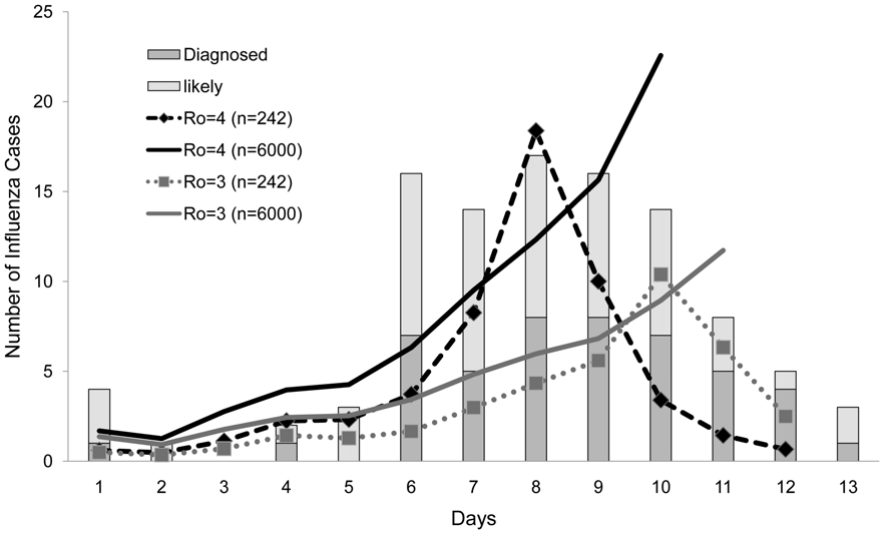
Determination of the model R_o_ from the SOPS epidemic curve. The number of observed and likely daily influenza cases (based on a pilgrims reported symptom onset date) at the SOPS clinic compared to the number of daily cases when decisions are made on the combination of the SOPS Outbreak case definition and a POCT result and using different R_o_ values. The likely epidemic curve represents the observed cases and the additional cases that would have resulted if all individuals presenting were tested. The R_o_ values are modeled on a closed population of 242 (the number of pilgrims presenting to the SOPS clinic) and 6000 (the minimum number of pilgrims accommodated at the SOPS).

Utilizing the SOPS Outbreak case definition followed by a POCT result, model simulations were performed to validate adopted influenza characteristics against the observed and likely epidemic curves. Likewise, the optimal R_o_ was established (Figure 3).

Subsequent simulations were performed on a hypothetical population of 6,000 naïve individuals using a R_o_ of 4.

### Model Simulations

**Figure 4:**
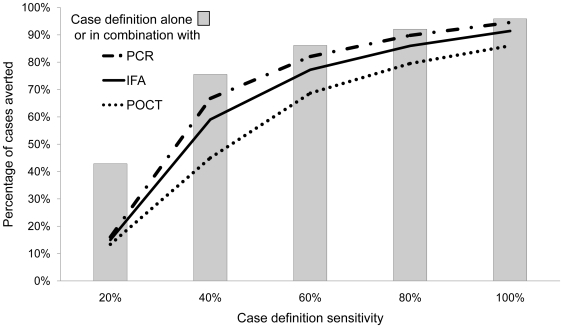
The impact of case definition sensitivities and a laboratory test on the number of influenza cases averted. The proportion of influenza cases averted at 17 days compared to no intervention, when isolation of influenza cases is based on increasing case definition sensitivities alone or in combination with a laboratory test.

In this model, isolation of patients based on a hypothetically 100% sensitive clinical case definition alone would result in 5% of the total population being infected by the end of 17 days. A reduction in case definition sensitivity corresponds with an increase in the number of infected individuals. When using coryza, the CDC or NSW case definitions, 12%, 29% and 51% of the total population would be infected with influenza at the end of 17 days, respectively. To avert greater than 50% of influenza cases at 17 days compared to no intervention, a case definition threshold of 27% was required (Figure 4). No case definition was able to terminate the outbreak.

As no laboratory test can be 100% sensitive, the infection control strategy guided by a combination of a case definition and any laboratory test will result in a greater proportion of the total population being infected with influenza at the end of 17 days compared to isolation of individuals using a case definition alone. In combination with a hypothetical 100% sensitive clinical case definition isolating individuals based on an immediate PCR result translated in an additional 2% of the total population being infected with influenza at the end of 17 days compared to isolating on symptoms alone. This increased to an additional 4% and 10% when isolation resulted from the combination of a 100% sensitive case definition and the less sensitive tests of an IFA or on-site POCT respectively. Similarly, for any combination of a case definition (with a sensitivity >20%) an immediate PCR testing would result in the least number of additional cases compared to IFA or POCT (Figure 4) with the maximal difference between the tests occurring at case definition sensitivities between 20% and 60%.

**Figure 5:**
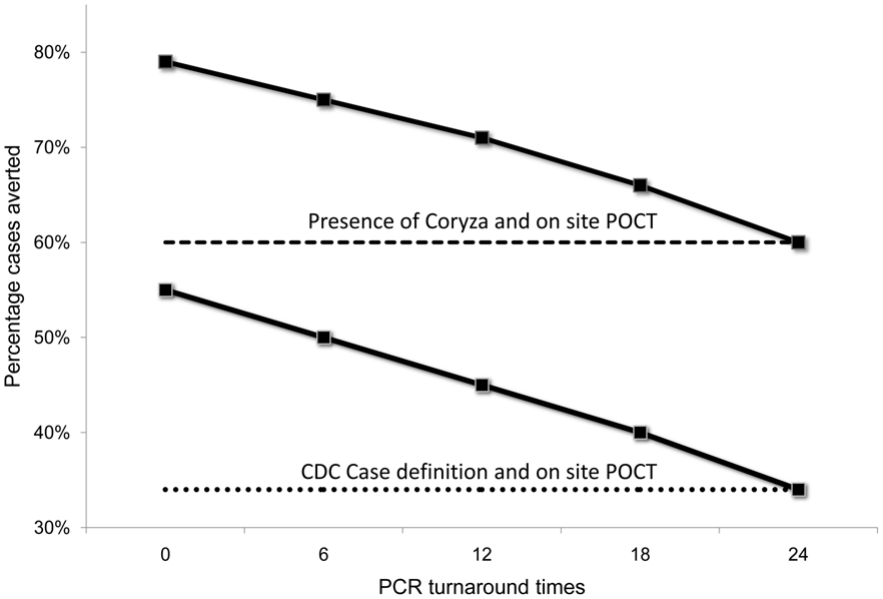
The impact of PCR TAT on the number of influenza cases averted. The percentage of influenza cases averted at day 17 based on PCR TAT in combination with either the presence of coryza or the CDC case definition compared to no intervention. The dotted lines represent the percentage of cases averted for the combination of the presence of coryza or the CDC case definition and a POCT. (With isolation of patients only after the receipt of a positive PCR result).

When PCR TATs were included in the model an increase in the total number of influenza cases resulted with increasing TATs (i.e. 6 to 24 hours). At day 17, a PCR with a TAT of 24 hours averted the same number cases compared to an on-site POCT (Figure 5). Similarly, an IFA test was equivalent to a PCR test with a 12 hour TAT. These associations remained stable, provided that clinical case definitions were above 20%.

**Figure 6:**
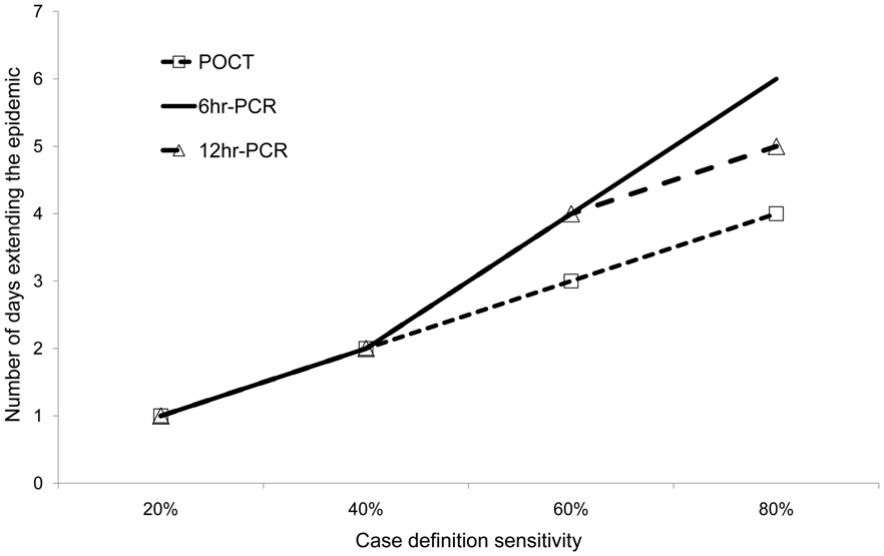
The value of different laboratory tests in extending an influenza outbreak. The number of additional days the outbreak is extended using a combination of a laboratory test and case definition compared to no intervention. IFA and PCR with a TAT of 24 hours were equivalent to the PCR with a 12 hour TAT and the POCT respectively.

Despite the apparent differences between the performance of laboratory tests at day 17, only small differences in delaying the peak of the outbreak (i.e. shifting the epidemic curve to the right) was achieved. A PCR with a 6 hour TAT would only result in extending the epidemic for an additional 1 or 2 days compared to a 12 hour PCR or an on-site POCT respectively, provided that the case definition sensitivity was above an 80% threshold (Figure 6).

**Figure 7:**
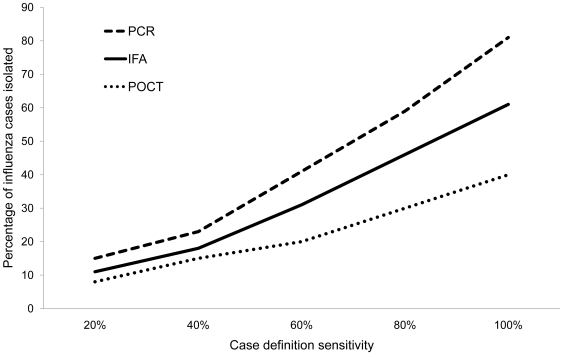
The role of laboratory testing in detecting true influenza cases. The percentage of influenza cases identified at day 17 based on the combination of a case definition and laboratory test compared to no intervention.

The major difference between the laboratory testing strategies is in the number of influenza cases potentially identified and thus amenable to an intervention. It also enables an intervention to be delivered to a smaller proportion of those meeting the case definition. In addition, antiviral prophylaxis would be more appropriately assigned to “true” contacts. PCR based laboratory confirmation allowed the isolation of the greatest proportion of symptomatic cases (Figure 7). For any case definition the number of symptomatic cases isolated was equivalent for all PCR TATs. With increasing case definition sensitivity, a larger proportion of symptomatic cases were isolated irrespective of laboratory test.

The effect of increasing the susceptible population (12000 to 1 million) using the most sensitive case definition (presence of coryza) with the addition of a laboratory test at day 17 was examined. The relative impact of clinical case definitions with or without the addition of laboratory testing remained unchanged irrespective of the size of the population. In addition, the impact of a PCR with a TAT of 24 hours was equivalent to an on-site POCT in the number of averted cases irrespective of population size.

Simulations using differing influenza characteristics revealed that the R_0_ had the biggest impact on the total number of infected individuals at the end of 17 days. With decreasing R_0_ values, the number of cases averted by any clinical case definition and laboratory test decreased. With R_0_ values greater than 4, no discernable differences between any case definition with the addition of any testing modality could be found. The associations between on-site POCT and PCR delayed by 24 hours remained at lower R_0_ values. Similarly, no difference was observed if the estimated prevalence was altered based on minimum and maximum attack rates in the untested population.

## Discussion

A decision tree analysis was undertaken to review the impact of different ILI case definitions and different laboratory testing methods on a larger outbreak, as might occur with other mass gatherings or during the very early phases of a pandemic. This was based on data prospectively collected from an outbreak of human influenza during WYD in 2008.

From our model simulations, the greatest number of ILI cases could be averted when isolation of individuals during an outbreak is based solely on a clinical case definition. Nevertheless, no case definition threshold (including a hypothetical 100% sensitive case definition) was found that would result in termination of the outbreak as recognition of asymptomatic infectious influenza cases remains problematic. Thus, the net impact of any case definition depends on its ability to delay the peak of the outbreak and to obtain more time for mobilization of alternative disease control strategies such as vaccination or antiviral prophylaxis. However, in our setting, if any of the previous published clinical case definitions were used, only a minimal (≤2 days) extension of the outbreak would have occurred. The “optimal” case definition (the presence of coryza) would extend the modeled outbreak by 8 days. Whether such a high case definition sensitivity can be achieved, especially in the early phases of an outbreak where symptomatology is uncertain and potentially variable in the different age groups, remains uncertain [6], [7].

The addition of a laboratory tests to any case definition would result in a greater number of influenza cases detected at day 17 compared to isolating patients based on symptoms alone, as no laboratory test has a 100% sensitivity. This effectively reduces the clinical case definition sensitivity by between 10% and 20% which in turn results in an increase in the proportion of infected individuals and a reduction in the number of additional outbreak days. In the ideal scenario of a 100% sensitive clinical case definition, PCR would have the highest utility in preventing further influenza cases. However, this is reliant on the PCR result being available immediately, a situation rarely achievable in clinical practice when factors such as specimen transportation and laboratory processing are taken into account. This was demonstrated during the WYD outbreak, where the greatest delay was due to specimen transportation to the laboratory. Even with an efficient courier system, it is likely that NAT would require a minimum of 6 to 12 hours for completion. With extended TATs, the utility of PCR is reduced, such that 12 and 24 hour PCR TATs are equivalent to IFA and on-site POCT respectively in terms the number of influenza cases averted.

What then is the utility of the various laboratory testing modalities? A laboratory test not only excludes falsely identified influenza cases due to a poorly specific case definition but also identifies true influenza cases who are suitable for an intervention, such as isolation or use of antiviral treatment. Therefore, use of a laboratory test is a balance between an increase in number of cases and the number requiring an intervention. The effectiveness of the subsequent intervention is in turn directly related to testing TAT and thus immediate less sensitive tests may have greater utility (on-site POCT) than highly sensitive but delayed tests (PCR with 12–24 hour TAT).

Changing the influenza attack rate significantly impacted on the ability of testing algorithms to ameliorate the epidemic. Most published models of an influenza pandemic used relatively low attack rates (R_o_<3) [4], [19], [24], [25]. Although, our attack rate (R_o_ 4.0) may seem high, much larger attack rates (R_o_ 7.5–10.6) in closed communities have been documented [22] including an R_o_ of 20 in a boarding house outbreak during the 1918 influenza pandemic [21]. Despite these concerns it was evident from our simulations that for any specific R_o_ value, the clinical case definition remains the most influential factor in the number of cases averted. Similarly, delayed PCR results (24 hours) and on-site POC testing remain equivalent in their performance.

Although our outbreak may not mirror an influenza pandemic, similar strategies using the same influenza characteristics have been applied to predict the behavior of the pandemic [4], [14], [22], [26], [27]. Furthermore, the principles used for determining the best laboratory and/or case definition is linked to the stage of the pandemic with highly sensitive rapid testing recommended in Australia (e.g. NAT) for the DELAY and CONTAIN stages [27]. This requires building laboratory surge capacity with reagent stockpiling, infrastructure acquisition and staff training. Logistics of specimen transportation laboratory processing and result reporting must also be addressed [26]. Our results suggest that this may not be an effective or appropriate strategy. In addition, decisions based solely on the combination of an individual's symptoms and laboratory tests (i.e. the absence of any other disease control strategy) would result in the relentless spread of the pandemic.

Our analysis has several potential limitations. Some of assumptions used in our model were based on published epidemiological studies. The heterogeneity of published evidence reflects different clinical case definitions, sample sizes, timing of sample collection, transmissibility of different influenza strains, the incidence of influenza and local healthcare practices including influenza vaccination. Also, our estimates could be affected by a publication bias as most studies originate from developed countries and may not be applicable to all populations. The model is constrained by a static population in contrast to the dynamic nature of pilgrims' movements to and from the various accommodation venues. This may affect not only the exposure probabilities but also the viral dynamics (e.g. R_o_). Previous vaccination or history of prior influenza infections were not included in the model and could similarly affect infection dynamics and exposure probabilities across the different simulations. This may have altered the magnitude of our findings but would not affect the overall associations or direction of our conclusions. Interventions in the model (i.e. isolation of individuals) were solely dependent on meeting the clinical case definitions and/or positive laboratory test results. This is often not the case during outbreaks as decisions are frequently multi-factorial (i.e based on known epidemiological linkages and the available resources). In addition, factors for these decisions tend to change over time because of varying disease patterns and logistics of managing large numbers of localized cases. Finally, the closure of the on-site clinic corresponded with the closure of the SOPS as an accommodation venue with further cases of influenza certainly occurring in pilgrims. It remains unknown whether these possible additional cases would have altered the performance of the various tests and/or case definitions. Symptoms such as myalgia and/or fatigue were not captured and may likewise have altered test performance if included. Despite all these limitations, the aim of our decision-analytic experiments was to improve the quality of decisions made in the face of uncertainty and incomplete information. As our model closely reflected the actual WYD 2008 outbreak data, it is likely that our assumptions are valid and that our findings can be generalized to larger outbreaks.

In conclusion, the impact of ILI case definitions and laboratory testing strategies modeled on data from the WYD 2008 outbreak demonstrated the critical role of specific clinical case definitions and the limited utility of additional laboratory interventions in containment of influenza during mass gatherings, seasonal epidemic and possibly pandemic influenza. Social distancing strategies guided by highly sensitive case definitions and epidemiological data are more likely to limit the spread of influenza during mass gatherings. Cost-benefit analyses of laboratory diagnostic strategies for influenza are required along with continuous re-evaluation of case definitions and testing strategies during both seasonal and mass gathering influenza outbreaks.
